# Direct cell-to-cell transmission of respiratory viruses: The fast lanes

**DOI:** 10.1371/journal.ppat.1007015

**Published:** 2018-06-28

**Authors:** Nicolás Cifuentes-Muñoz, Rebecca Ellis Dutch, Roberto Cattaneo

**Affiliations:** 1 Department of Molecular and Cellular Biochemistry, University of Kentucky College of Medicine, Lexington, Kentucky, United States of America; 2 Department of Molecular Medicine and Virology and Gene Therapy Graduate School Track, Mayo Clinic, Rochester, Minnesota, United States of America; Mount Sinai School of Medicine, UNITED STATES

## Introduction

**Fig 1:**
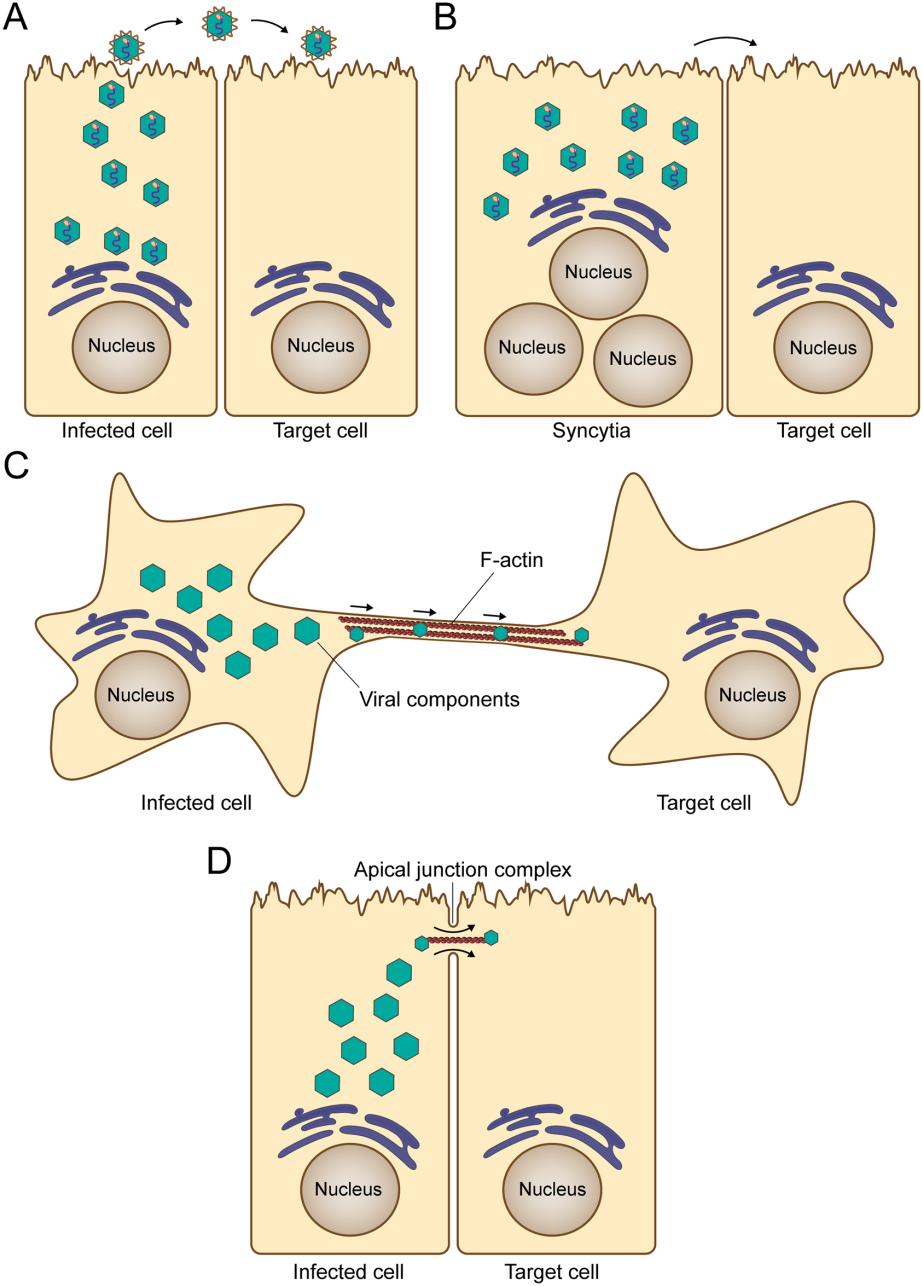
Classical and new mechanisms of spread by respiratory viruses. A) The canonical route of virus spread involves the release of completely assembled viral particles to the extracellular environment for subsequent infection of target cells. B) The formation of syncytia involves the fusion of infected cells with adjacent target cells and remains an important mechanism of direct cell-to-cell spread of viral components. This mechanism of spread has been reported for influenza, HMPV, PIVs, RSV, and MeV, among others. C) Intercellular extensions connect two distant cells to facilitate transport of viral components, and their formation requires F-actin polymerization. This mechanism of viral spread has been reported in immortalized lung epithelial cells for influenza, PIV5, HMPV, and RSV. D) Intercellular pores connect two adjacent cells, allowing flow of viral components between infected cells and target cells. This mechanism of spread was described for MeV in well-differentiated primary cultures of human airway epithelial cells. F-actin, filamentous actin; HMPV, human metapneumovirus; MeV, measles virus; PIV, parainfluenza virus; PIV5, parainfluenza virus 5; RSV, respiratory syncytial virus.

Virus particles protect genomes from hostile environments within and outside the host, eventually delivering these genomes to target tissues to initiate infection. Complex processes requiring significant energy and time are necessary to assemble these virus particles, but only a small portion of released virus will successfully infect new target cells (Fig 1A). While the science of virology has developed based on the isolation and purification of viral particles, it is becoming increasingly clear that direct cell-to-cell transmission of viruses and/or viral components is also highly relevant [1,2].

Direct cell-to-cell spread of infections has several advantages. The first is efficiency: genomic cargo is delivered directly to cells rather than being randomly released into the environment. The second is speed: appropriation of cellular protein trafficking infrastructure allows faster spread within tissues. The third is barrier avoidance: intrinsic immunity and other barriers interfering with entry or post-entry steps in target cells can be bypassed. The fourth is humoral immunity evasion: limited exposure time to the extracellular space allows evasion of neutralizing antibodies.

## What are “fast lanes” and why are they needed in the respiratory tract?

From the speed and efficiency perspective, we refer to the infrastructure that viruses use for rapid cell-to-cell spread as “fast lanes.” Viruses do not build fast lanes. Instead, they modify or connect existing host-cell trafficking and cytoskeletal infrastructure. In this sense, different categories of fast lanes exist that vary in their complexity. One of the best characterized is the formation of giant multinucleated cells, called syncytia, which result from membrane fusion and cytoplasmic content mixing between infected cells and neighbor cells [1] (Fig 1B). A recently described intracellular fast lane consists of strategically located openings that allow intercellular exchange of cytoplasm and viral genomes [3]. Exchange of viral components has also been recently shown to occur through filamentous structures that extend towards distant cells [4–6]. Other fast lanes, while forming below the cell surface, push released particles to “surf” towards noninfected cells [7]. Moreover, some viruses take advantage of structures built by circulating cells to communicate, such as the immunological synapse, to spread more rapidly [2]. We focus here on the recently described fast lanes employed by different respiratory viruses that may allow rapid spread in the respiratory tract.

Respiratory viruses comprise a large group of pathogens that spans different viral families. Among those, we refer to the orthomyxovirus influenza virus, paramyxoviruses such as measles virus (MeV) and parainfluenza viruses (PIVs), and the pneumoviruses respiratory syncytial virus (RSV) and human metapneumovirus (HMPV). All of these pathogens exploit the most common route of viral entry into humans: the epithelial lining of the airways. However, the respiratory tract environment represents a challenge for released particles: A mucociliary blanket lines the tract, trapping particles and carrying them back to the throat for destruction. In addition, mucus can contain antibodies that neutralize particles. Moreover, the lowest portion of the tract that lacks cilia and mucus is safeguarded by macrophages that destroy particles. Thus, mechanisms that allow viral spread within the airway epithelia without particle release would be highly advantageous, and recent studies suggest that some respiratory viruses have indeed developed alternative means of spread.

## Why are intercellular extensions and intercellular pores fast lanes?

We generically refer to the first type of fast lanes as intercellular extensions (Fig 1C), though subtle differences in the nature of these extensions have been reported for different pathogens. Influenza virus and PIV5 infection in cell culture result in the formation of intercellular extensions resembling tunneling nanotubes (TNTs), which project from the cell body to reach distantly located cells. Openings at the end of these structures can be used to transfer viral components, including genomes and proteins, between infected lung epithelial cells and neighbor cells [4,8]. This unconventional mechanism of spread allows influenza virus to disseminate infection even in the presence of neutralizing antibodies or the neuraminidase inhibitor oseltamivir [4,8]. The use of TNTs as a mechanism for efficient cell-to-cell spread has recently been reported for other unrelated viral pathogens including human immunodeficiency virus-1 [9], porcine reproductive and respiratory syndrome virus [10], and pseudorabies virus [11]. HMPV infection of human bronchial epithelial cells also results in formation of intercellular extensions that facilitate direct cell-to-cell spread [5]. Similar to influenza, HMPV cell-to-cell spread occurs in the presence of neutralizing antibodies and in the absence of the appropriate attachment factors [5]. It is also notable that RSV infection induces formation of intercellular extensions in human alveolar A549 epithelial cells, and that virus spread in these cells depends on actin polymerization [6]. Both HMPV- and RSV-induced intercellular extensions resemble filopodial structures, which have structural differences compared with TNTs [9,12]. The use of filopodia as a mechanism of efficient spread has been described for alphaviruses [13,14], asfarvirus [15], and the retrovirus human T-lymphotropic virus [16].

Studies in well-differentiated primary cultures of human airway epithelial cells infected with MeV have identified a second type of fast lane: intercellular pores (Fig 1D). In these cultures, MeV infectious centers form more rapidly and become larger than those of RSV, Sendai virus, or PIV5 [17]. While visible syncytia do not form after MeV infection, the cytoplasm of infected cells suddenly flows into adjacent columnar cells through openings that form on the lateral surfaces at locations corresponding to the apical junction complex, where the MeV epithelial receptor nectin-4 is located [17,18]. To our knowledge, similar structures for efficient cell-to-cell spread have yet to be described for any other virus.

While the structures involved in direct cell-to-cell spread can be structurally distinct, they do share common characteristics. First, cytoskeletal filaments are used for intracellular transport through these structures to an opening that allows viral components to enter noninfected cells, likely at a location providing immediate access to the transport infrastructure of the target cells. Second, the complete assembly of virions is not needed, and direct cell-to-cell transfer of viral genetic material, proteins, nucleocapsids, or replication bodies is most likely occurring. Finally, disruption of intercellular extensions or intercellular pores by different means leads to reduced and/or less efficient viral spread. For these reasons, we consider intercellular extensions and pores fast lanes.

## How are intercellular extensions and intercellular pores built?

The formation of TNTs induced by influenza virus and PIV5 in lung epithelial cells involves drastic changes in the host cell cytoskeleton, requiring both filamentous actin (F-actin) and microtubule dynamics [4,8]. Though TNTs are present in noninfected cells and are thought to be involved in the transfer of vesicles and organelles [19], they occur more frequently in infected cells. HMPV induces the formation of intercellular extensions resembling filopodia and also induces networks of branching filaments that project from both the cell body and the extensions [5]. Filopodia formation in noninfected cells is associated with diverse functions including migration, chemoattractant sensing, and adhesion to the extracellular matrix [20]. Similar to influenza-induced TNTs, HMPV-induced intercellular extensions are more abundant and also longer in infected cells. F-actin dynamics and the Rho GTPases Cdc42, Rac1, and RhoA, which regulate actin cytoskeleton dynamics and are involved in cell-to-cell fusion and syncytia formation [21], contribute to the formation of HMPV intercellular extensions, with Cdc42 and Rac1 having a more significant impact than RhoA. Surprisingly, a potential role in actin nucleation for the viral polymerase cofactor phosphoprotein (P) was also found, suggesting novel functions for this protein beyond its role in replication [5]. The RSV fusion protein and cellular actin-related protein 2 (ARP2) were shown to be involved in the formation of filopodial structures [6]. ARP2 is a key component of the ARP2/3 complex, which plays a crucial role in actin nucleation and is induced by Cdc42 through the activation of the Wiskott–Aldrich syndrome protein (WASp). As mentioned above, viruses do not unilaterally build fast lanes, but consistently, intercellular extensions are more abundant in infected cells. This suggests that some viral components must influence the dynamics of the cell cytoskeleton in order to spread more efficiently.

Examination of the cellular components required for MeV-induced pore formation in epithelial layers has focused on the role of the cellular MeV receptor, nectin-4. To assess whether the connection of nectin-4 with the actin cytoskeleton is required for efficient MeV transmission, this connection was interrupted using two different approaches: by small interfering RNA (siRNA) knock-down of afadin, the cellular protein that tethers nectin-4 to actin, and by using nectin-4 deletion mutants. Both operations resulted in reduced spread, indicating that MeV relies on both ends of its receptor for efficient spread in airway epithelia: the tip of the nectin-4 extracellular domain provides the binding site, while the cytoplasmic tail connects to the cytoskeleton through afadin. This connection is key to very rapid spread, because the cytoskeleton of columnar cells is anchored to either side of the apical junction complex. Importantly, in this system, rapid lateral spread occurs without cytopathic effects, and transepithelial resistance remains intact [22]. This suggests that MeV intercellular pores do not expand, probably because the cytoskeletal connection of the apical junction complex constrains them. Additional cellular factors likely contribute to opening of the intercellular pore, including proteins from the Rho GTPases family, but these remain to be explored.

## The fast lanes: What we don't know

**Fig 2:**
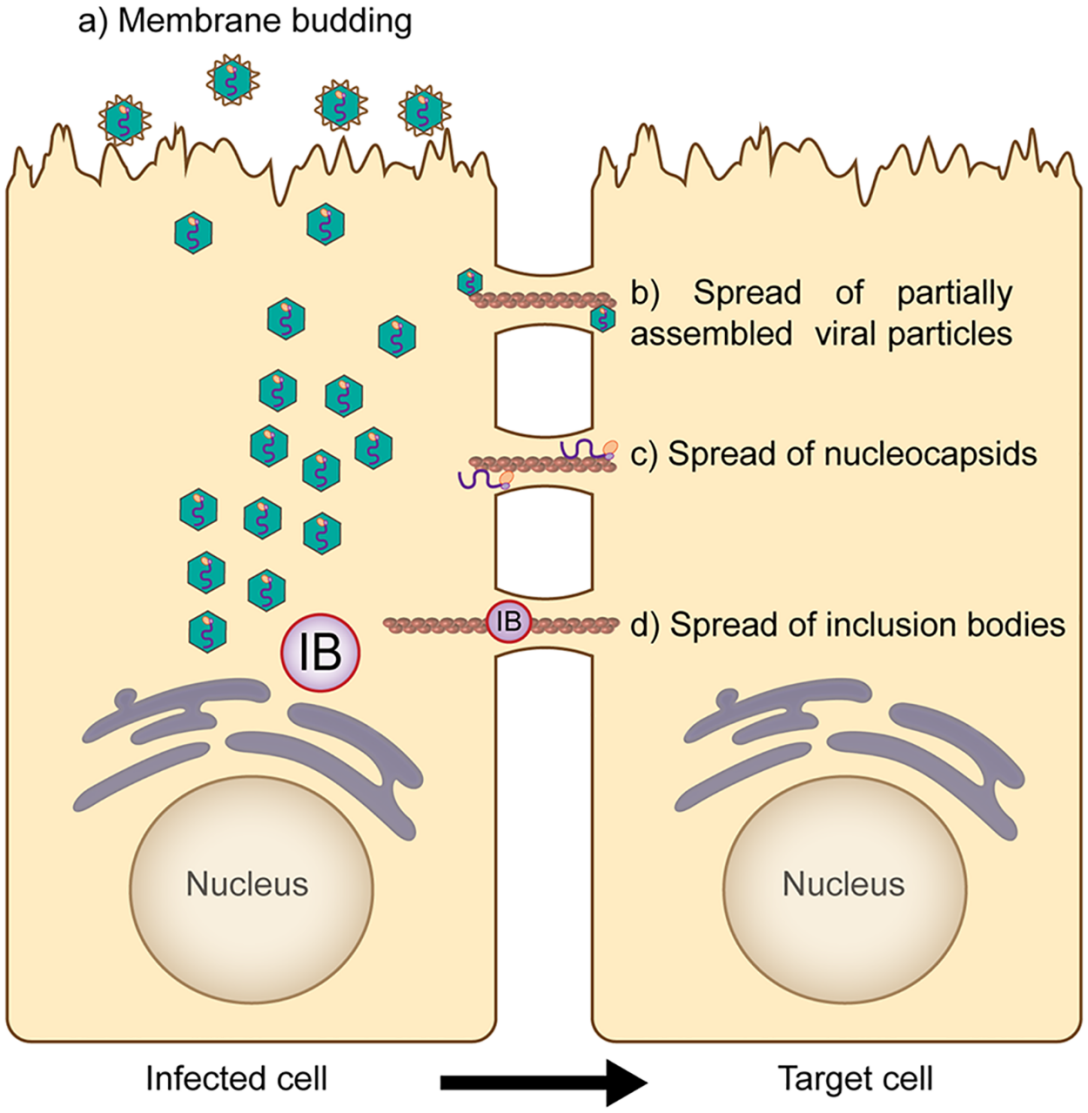
Different mechanisms of viral spread that may occur in respiratory cells through fast lanes. From infected cells, completely assembled viral particles are released independently through membrane budding (a), which likely plays a major role in interhost spread of infection. Alternatively, viral infection can be spread intercellularly through fast lanes. This route of infection does not require complete assembly of viral particles and, hypothetically, spread might occur in the form of partially assembled viral particles (b), nucleocapsids (c), or inclusion bodies (d). These mechanisms might not be exclusive, as membrane budding and direct cell-to-cell spread might occur simultaneously in infected cells.

Fast lanes may be in play at other mucosal surfaces, and the work on respiratory viruses summarized here may facilitate their identification and characterization. Recent findings that multiple respiratory viruses promote cell-to-cell spread through actin-based structures suggest the involvement of the cytoskeleton in pathogenesis, but in vivo studies have remained elusive. The use of 3D airway epithelial tissue models with MeV has allowed demonstration of the actin-dependent formation of intercellular pores wide enough to allow the passage of viral nucleocapsids [17]. However, the formation of the intercellular extensions induced by influenza, HMPV, and RSV has not been shown in 3D systems or in vivo, and the mechanistic relationship between pores observed in airway models and intercellular extensions observed in 2D models remains unclear. In addition, several important features of these structures are not yet fully understood. For example, though intercellular connections induced by influenza were shown to be open ended, thereby resembling TNTs [4,8], the open-ended nature of filopodia-like structures induced by RSV and HMPV remains to be addressed. Furthermore, the critical question of what exactly is being transferred through intercellular pores or extensions has not been resolved. Viral proteins and genomic RNA have been observed within these structures, but it seems unlikely that completely assembled virions, generally released through membrane budding (Fig 2a), might be transferred through intercellular pores or extensions. Instead, we hypothesize that direct cell-to-cell spread of infection might occur in the form of partially assembled viral particles (Fig 2b) or nucleocapsids (Fig 2c). Interestingly, structures similar to inclusion bodies, which recent studies strongly suggest are organelles involved in replication for several respiratory viruses [23,24], have been observed moving through intercellular extensions [5]. The potential transfer of a replicative inclusion body from one cell to another (Fig 2d) would allow the virus to bypass the first critical hours of infection, and examination of this intriguing hypothesis could alter our understanding of viral spread.

A deeper understanding of the balance and control of both cell-free and cell-associated viral spread warrants future research. It is possible that the formation of intercellular extensions and pores may benefit an infected cell by facilitating exploration of the environment and cell-to-cell communication. Interestingly, the fast lanes discussed here may promote mass transport rather than traffic of individual genomes. This would change the dynamics of replication while also releasing bottleneck pressure on genome evolution. In other words, virus genome populations moving through mass transit in host tissue are bound to differ significantly from genome populations selected by repeated passage of individual particles on transformed cells. A host-organism–oriented understanding of viral replication will improve our approaches in the search for new antiviral strategies and vaccines, as no reports have addressed how the new mechanisms of spread affect the antiviral immune response.
